# Evaluation of Insecticide Resistance in *Aedes albopictus* Population from Algiers, Algeria

**DOI:** 10.3390/insects17070696

**Published:** 2026-07-04

**Authors:** Rym Bouledroua, Amira Nebbak, Nicolas Gomez, Zakaria Abdellahoum, Mustapha Mounir Bouhenna, Slimane Boukraa, Khaldoun Bachari, Philippe Parola, Sébastien Briolant, Lionel Almeras

**Affiliations:** 1 Unité Mixte de Recherche Risques Infectieux Tropicaux et Microorganismes Emergents, Assistance Publique-Hôpitaux de Marseille, Service de Santé des Armées, Aix Marseille Université, 13005 Marseille, France; rymbouledroua23@gmail.com (R.B.); nico13dna@hotmail.com (N.G.); philippe.parola@univ-amu.fr (P.P.); 2 Institut Hospitalo-Universitaire (IHU)-Méditerranée Infection, 13005 Marseille, France; 3 Centre de Recherche Scientifique et Technique en Analyses Physico-Chimiques (CRAPC), Tipaza 42004, Algeria; amiranebbak@yahoo.fr (A.N.); m.m.bouhenna@gmail.com (M.M.B.); bachari2000@yahoo.fr (K.B.); 4 Unité Parasitologie et Entomologie, Département Risques Vectoriels, Institut de Recherche Biomédicale des Armées (IRBA), 13005 Marseille, France; 5 Laboratoire de Protection des Végétaux en Milieux Agricoles et Naturels, Département de Zoologie Forestière et Agricole, Ecole Nationale Supérieure Agronomique (ENSA), Algiers 16004, Algeria; zabdellahoum@gmail.com (Z.A.); slimane.boukraa@edu.ensa.dz (S.B.)

**Keywords:** *Aedes albopictus*, insecticide resistance, *kdr* mutations, metabolic resistance, esterases, vector control, Algeria

## Abstract

Since its first detection in 2010, *Aedes albopictus* has spread across northern Algeria, where vector control relies on the use of chemical insecticides. This study aimed to evaluate the susceptibility of *Ae. albopictus* populations from Algiers to commonly used larvicides and adulticides, as well as to characterize the underlying resistance mechanisms. Eggs were collected from three sites in Algiers. The susceptibility of larvae to temephos and *Bacillus thuringiensis israelensis* (*Bti*), as well as that of adults to permethrin, deltamethrin, malathion, and bendiocarb was evaluated using WHO bioassays. Genotyping of knockdown resistance (*kdr*) mutations was performed via PCR and sequencing. Metabolic resistance mechanisms were investigated using CDC bottle bioassays. The larvae were found to be susceptible to temephos and *Bti*. Bioassays on adults demonstrated susceptibility to deltamethrin, suspected resistance to permethrin, and resistance to malathion and bendiocarb. Genotyping revealed low frequencies of heterozygous *kdr* mutations (V1016G, I1532T, F1534C/S). Synergist assays highlighted the key role of esterases in malathion resistance, a minimal involvement of glutathione S-transferases and an unexpected antagonistic effect of cytochrome P450 monooxygenases. Although larvicides remain effective, resistance to organophosphates, carbamates, as well as suspected resistance to permethrin, has been detected. Esterase-mediated metabolic resistance and *kdr* mutations may contribute to this profile. These findings highlight the need for resistance monitoring and integrated vector control to ensure sustainable control of *Ae. albopictus*.

## Simple Summary

The Asian tiger mosquito, *Aedes albopictus*, has spread rapidly throughout northern Algeria and poses a growing threat to public health, as it can transmit viruses such as dengue, chikungunya, and Zika. Mosquito control in Algeria relies on the use of chemical insecticides, but repeated use can make mosquitoes less susceptible to these products. Until now, no study had sought to determine whether Algerian populations of this mosquito were developing resistance to insecticides. We tested mosquitoes collected in Algiers to evaluate the effectiveness of products commonly used against immature and adult stages. Our results showed that the products used to eliminate larvae remain highly effective. In contrast, some insecticides used against adult mosquitoes proved less effective: clear resistance was detected for certain compounds, while others showed early signs of resistance. We also identified biological changes enabling mosquitoes to survive exposure to insecticides, notably increased activity of detoxification processes and the presence of early genetic modifications associated with resistance. These results provide the first evidence of the emergence of insecticide resistance in *Aedes albopictus* in Algiers. Regular monitoring and more sustainable vector control strategies—particularly reducing reliance on a single type of insecticide—will be essential to protect public health and maintain effective control of this arbovirus vector.

## 1. Introduction

Algiers, the capital of Algeria, is located on the southern shore of the Mediterranean Sea. It has a large seaport on the Mediterranean Sea, used for economic activities, fishing and passenger transport, as well as an international airport serving several countries around the world. The city of Algiers also contains a large wetland of 842 ha, known as Lake of Reghaia, which serves as an important stopover for migratory birds coming from the Mediterranean and the Sahara Desert [1]. In addition, it has a domestic airport serving the country’s southern provinces. This makes the city of Algiers a potential gateway for the introduction of viruses transmitted by mosquitoes of the genus *Aedes*.

*Aedes* (*Ae*.) *albopictus* Skuse, 1894, the tiger mosquito native to Southeast Asia, is one of the world’s most invasive species and has now established populations on all five continents [2]. *Aedes albopictus* is not only an invasive and harmful species, but it also transmits several human pathogens, including arboviruses such as the dengue, chikungunya, and Zika viruses, making it a major threat to public health [3]. In Algeria, the presence of *Ae*. *albopictus* was confirmed for the first time in August 2010 in Larbaa-Nath-Irathen (Tizi-Ouzou province), when a live female, partially engorged with blood, was captured as part of an entomological program originally intended to study sandflies [4]. Four years later, in the same province, two adult specimens of *Ae*. *albopictus* were captured in the village of Illoula Oumalou [5]. In 2015, the presence of this species was confirmed in Aïn Turk, in the wilaya of Oran, where all stages of development were observed [6]. The following year, an entomological study conducted in the Bir-Khadem district of Algiers revealed the presence of *Ae*. *albopictus* through the collection of 57 specimens, providing the first evidence of the species’ introduction into the Algerian capital [7]. Since then, this invasive mosquito species has spread rapidly to other regions of northern Algeria, notably to the province of Annaba in 2017 [8], and then to Jijel and Skikda in 2019 [9]. Recently, numerous studies have confirmed its rapid spread through the colonization of natural and artificial habitats starting from the northeastern part of the country [10,11,12]. Currently, *Ae*. *albopictus* is present throughout northern Algeria and is considered the second most abundant species after *Culex pipiens* [11]. Its presence has caused considerable inconvenience to the population, but has also increased the risk of infection by viruses transmitted by mosquitoes of the genus *Aedes* [7]. Since 2017, healthcare facilities have reported several cases of patients seeking medical care for itching and secondary infections caused by mosquito bites [6,13].

A recent study conducted in Algeria revealed the circulation of several medically significant arboviruses transmitted to humans by mosquitoes [14]. Among these, the chikungunya virus (CHIKV), which belongs to the genus *Alphavirus* (family *Togaviridae*), is primarily transmitted by mosquitoes of the genus *Aedes*, particularly *Ae*. *albopictus* [15]. The detection of CHIKV in mosquitoes collected in Algeria suggests a real risk of local transmission of this arbovirus. Furthermore, reports of imported dengue cases in areas colonized by the *Ae*. *albopictus* vector indicate that the risk of arboviruses epidemics in humans is not merely hypothetical [16]. These observations underscore the importance of strengthening entomological surveillance and developing effective vector control strategies to reduce the burden of these diseases and prevent outbreaks [17].

Vector control relies on targeted interventions aimed at reducing larval and adult populations [18]. For larvae, control measures consist primarily of managing breeding sites, either by eliminating them or by applying biological and chemical treatments [19]. For adult mosquitoes, control relies primarily on the use of insecticides applied directly to the environment—either by spraying or through surface treatments—to reduce mosquito density and their ability to transmit pathogens [20,21].

In Algeria, mosquito control relies primarily on the use of organophosphates and synthetic pyrethroids targeting both larvae and adults [22]. Treatments are applied regularly according to a fixed schedule throughout the year, with more frequent interventions in the spring and summer when mosquito density increases [23]. Consequently, despite intensified vector control and eradication efforts over the past decade, the invasive species *Ae*. *albopictus* has become permanently established in the country [24].

The treatments are sprayed or applied manually, particularly around homes, on stagnant water, and near garbage dumps, slaughterhouses and cellars [25,26]. Frequent and repeated application of insecticides exerts strong selective pressure, promoting insecticide resistance in mosquitoes through various mechanisms, such as (i) mutations at the target site (e.g., knock-down resistance (*kdr*) mutations in the voltage-gated sodium channel (*vgsc*) gene) [27,28], (ii) metabolic detoxification through increased activity of detoxification enzymes (e.g., cytochrome P450s monooxygenases, esterases, and glutathione S-transferases (GSTs)) [29], (iii) thickening of the cuticle to reduce insecticide penetration, and (iv) behavioral changes aimed at limiting contact with insecticides [30,31]. To date, few studies have assessed mosquito resistance to insecticides in the Maghreb (i.e., Algeria, Morocco, Tunisia), and most of these have focused on *Cx*. *pipiens sl*. [32,33,34,35]. However, no study has examined the susceptibility of *Ae*. *albopictus* to the main classes of insecticides in Algeria. Chemical pesticides, which are harmful to human health and the environment, are increasingly being replaced by biopesticides, as part of a “One Health” approach that emphasizes the importance of Integrated Vector Management (IVM) [36]. To this end, studies are needed to first clarify the extent of insecticide resistance in the vector species *Ae*. *albopictus* in Algeria, in order to promote integrated control strategies.

With this in mind, the objective of was to evaluate the susceptibility of *Ae*. *albopictus* populations in Algiers to pyrethroid, carbamate and organophosphate insecticides, as well as the associated resistance mechanisms. A carbamate-based insecticide was used in this study, even though it is not used in Algiers by the authorities responsible for vector control, as it could serve as an alternative to other insecticides in the event of resistance to pyrethroids and organophosphates. The phenotypic susceptibility of *Ae*. *albopictus* populations from Algiers to larvicides (*Bacillus thuringiensis israelensis* (*Bti*), temephos) and adulticides (permethrin, deltamethrin, malathion, bendiocarb) was assessed using standardized WHO tests [37]. In addition, the relationship between *kdr* genotypes and pyrethroid resistance was also evaluated and synergy tests were conducted to assess the contribution of metabolic resistance. Understanding the resistance mechanisms of *Ae*. *albopictus* is essential for developing effective and sustainable local vector control strategies, particularly in urban environments such as Algiers. This study aims to make vector control more effective through science-based decision-making by selecting insecticides suited to the local mosquito population.

## 2. Materials and Methods

### 2.1. Mosquito Populations and Sampling Sites

**Figure 1:**
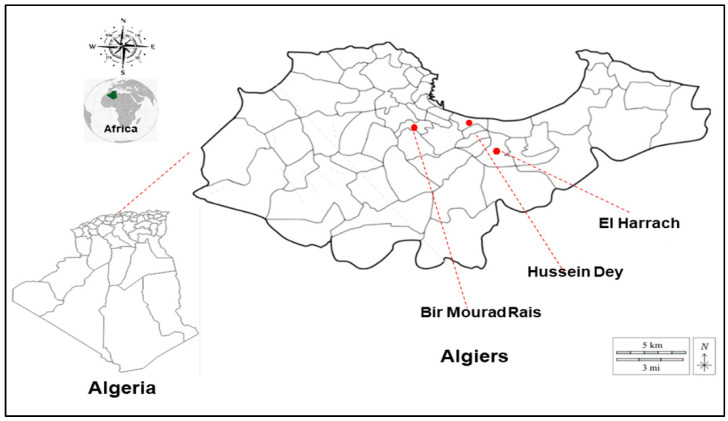
Geographic location of the three *Aedes albopictus* egg sampling sites in Algiers, Algeria. The main map shows the location of Algiers within Algeria, while the inset provides a magnified view of the province of Algiers, including its administrative districts.

*Aedes albopictus* eggs were collected in the field at three different locations in Algiers, Algeria, using ovitraps (Figure 1 and Figure S1, Table S1). Collection was carried out on private property with the owners’ consent. The ovitraps consisted of a black cylindrical container with a capacity of approximately 1300 mL (14 cm diameter, 12 cm in height), filled with tap water to two-third of its height. A 25 cm^2^ polystyrene square, floating and half-submerged, served as an oviposition substrate *Ae*. *albopictus* females. Trapping began in early September and continued until the end of October 2024. In total, approximately 2000 eggs were collected. These eggs, representing the founding generation (F0), were transferred from the Zoology Laboratory of the National Higher School of Agronomy of Algeria to the IHU-Infection Mediterranean center in France, under official import authorization (import permit no. ER 104-2024).

In addition to field-collected populations, eggs from a susceptible reference strain of *Ae*. *albopictus* (ATM-NJ95, generation F51 [NR-48979])—originating from Keyport, NJ, USA, in 1995 [38]—were provided by the Centers for Disease Control and Prevention (CDC) through BEI Resources, NIAID, NIH. This strain is fully susceptible to all major classes (pyrethroids, organophosphates, carbamates, organochlorines) [38] and was included in the study as a reference population for comparison during insecticide susceptibility tests.

### 2.2. Mosquito Rearing Procedures

Eggs from the three collection sites were then hatched separately in osmosis water. An equal proportion of larvae from each site was then mixed and reared on a standardized daily ration of fish food in 24 × 34 × 9 cm plastic trays, at a density of approximately 400 larvae per tray. Following pupation and emergence, 400 adult mosquitoes (males and females) were placed in 24 × 24 × 24 cm cages with continuous access to a 10% sucrose solution. Adult females were fed twice weekly with human blood supplied by the Etablissement français du sang (EFS), using an artificial feeding system (Hemotek Ltd., Blackburn, UK) equipped with a parafilm membrane. Access to human blood was based on an agreement concluded with the EFS (N° 4500557592_2025). All adult mosquitoes of the F0 generation were identified as belonging to the species *Ae*. *albopictus* using a morphological identification key [39]. Larvae and adults were reared under standardized conditions (28 °C, 75 ± 5% relative humidity, 12:12 h light–dark cycle) as previously described [27]. Larvicidal and adulticidal bioassays were performed on second-generation (F2) mosquitoes of the species *Ae*. *albopictus* originating from Algiers. The *Ae*. *albopictus* ATM-NJ95 strain, reared under the same conditions as previously described and without exposure to insecticides, served as a susceptible reference strain to assess susceptibility levels.

### 2.3. WHO Bioassays

#### 2.3.1. Larval Bioassays

The larvae of *Ae. albopictus* at the late third (L3) and early fourth (L4) stages were used for larvicide testing (Figure S2). Two larvicides were tested: the bioinsecticide *Bacillus thuringiensis israelensis* (*Bti*) (VectoBac AM65-52 strain, Edialux, Replonges, France), and the synthetic organophosphate temephos (Sigma-Aldrich, St. Louis, MO, USA). For each compound, seven distinct concentrations were tested to cover the full range of lethal responses from no effect to near-total mortality [38,40]. Concentrations of the compound ranged from 0.9375 to 60 µg/L for temephos and from 6.25 to 200 µg/L for *Bti*, with half-dilution between two points. The experimental groups were composed of 25 L3/L4 larvae of *Ae*. *albopictus*. These larvae were placed in 100 mL of solution, composed of 99 mL of tap water with the addition of 1 mL of different concentrations of insecticide solution. The control group (negative control) was composed of 25 L3/L4 larvae of *Ae*. *albopictus* of the same strain, placed in a solution of 99 mL of tap water and 1 mL of acetone (BDH Chemicals, VWR International, Leuven, Belgium). Four replicates of 25 larvae were used at each concentration in plastic cups per turn and three sets of experiments were performed independently. Mortality counts were recorded 24 h after exposure. The number of dead larvae was used to determine Lethal Concentrations (LC), including the median lethal concentration (LC_50_; concentration causing mortality in 50% of larvae) and LC_95_ values (the concentration causing mortality in 95% of larvae), to characterize and describe the relationship and dose–response curve according to standard procedures recommended by the World Health Organization (WHO). The experiments were replicated three times independently on both colonies, *Ae*. *albopictus* which was collected in the field in Algiers and the susceptible strain of *Ae*. *albopictus* ATM-NJ95. The *Ae*. *albopictus* ATM-NJ95 colony was used as a susceptible reference strain to assess susceptibility levels and calculate resistance ratios (RRs). Resistance was evaluated by calculating the RR_50_ ratio, obtained by dividing the LC_50_ of the tested population by the LC_50_ of the susceptible reference strain (RR_50_ = LC_50_ of the population/LC_50_ of the susceptible strain). A population is considered susceptible when RR_50_ < 5, moderately resistant when 5 ≤ RR_50_ ≤ 10, and resistant when RR_50_ > 10 [40,41].

#### 2.3.2. Adult Mosquito Bioassays

To assess adult susceptibility to insecticides used in public health, the bioassays (Figure S3) were carried out in accordance with WHO standard protocols [40], the global reference for insecticide resistance monitoring. Insecticide-treated papers provided by the WHO (Vector Control Research Unit, University of Science, Penang, Malaysia) were used at discriminant concentrations to assess the susceptibility profile of *Ae*. *albopictus* in Algiers to different classes of insecticides. Adult female mosquitoes 3–5 days old were exposed to a set of key insecticides representing the main chemical classes used in vector control programs, including pyrethroids type I and type II, permethrin (0.4%) and deltamethrin (0.03%), respectively, the organophosphate malathion (1.5%) and the carbamate bendiocarb (0.2%) (PESTANAL^®^, Merck, Darmstadt, Germany). For each insecticide tested at a discriminant concentration, approximately 25 adult female mosquitoes that had never taken a blood meal were carefully placed in exposure tubes lined with insecticide-impregnated papers. Four replicates of at least 25 specimens were performed for each concentration using plastic tubes, resulting in a total of at least 100 mosquitoes tested per insecticide per test series; three independent series were conducted. After one hour of exposure, the mosquitoes were transferred to clean holding tubes (i.e., observation tubes). A cotton swab soaked in a 10% sucrose solution was provided. Mortality was recorded 24 h after exposure. To assess resistance mechanisms, live and dead specimens were separated and placed individually in 96 well-plates, which were stored at −80 °C pending future analysis. Concurrently, two control tubes were used for each bioassay containing paper impregnated with silicon oil and acetone for pyrethroids, or olive oil and acetone for carbamates and organophosphates.

### 2.4. Genotyping of kdr Mutations

The abdomens of the selected mosquitoes were dissected and used for *kdr* genotyping. A total of 188 *Ae*. *albopictus* mosquitoes exposed to deltamethrin (0.03%) and permethrin (0.4%), were selected for this genotyping. For each insecticide, all surviving (i.e., resistant) mosquitoes were included, and dead specimens were randomly selected to reach a total of 94 per compound. Each specimen was individually homogenized using a TissueLyser II (Qiagen S.A.S., Courtaboeuf, France), and DNA was extracted according to the previously described method [27]. Nine *kdr* mutations, namely V410L, L982W, S989P, A1007G, I1011V, V1016G, T1520I, I1532T and F1534C/S, were genotyped. Three fragments of the *vgsc* gene were amplified by three separate polymerase chain reactions (PCR), using primer pairs designed with Geneious Prime software, version 2022.2.2 (Technelysium Pty Ltd., Tewantin, Australia). Details regarding the targeted codons, the primers used, and their final concentrations are summarized in Table S2. Each PCR amplification was performed in a 22 µL reaction volume using a master mix containing DreamTaq polymerase, buffer, 2 mM MgCl_2_, and deoxyribonucleotide triphosphates (dNTPs) (DreamTaq™ Green PCR Master Mix; Thermo Fisher Scientific, Illkirch, France). The master mix was supplemented with 3 µL of eluted DNA, and amplification was carried out using an Applied Biosystems™ Veriti™ 96-well thermal cycler (Applied Biosystems, Thermo Fisher Scientific, Foster City, CA, USA). The thermal cycling programs used for each primer pair are detailed in Supplementary Table S2. PCR products were separated on a 2% agarose gel stained with SYBR Safe™ (Thermo Fisher Scientific, Waltham, MA, USA) and visualized under ultraviolet light. Sequencing of positive PCR products was performed using a 3500 Series Genetic Analyzer for Sanger sequencing (Applied Biosystems^®^; Foster City, CA, USA). The sequences were aligned and compared with GenBank reference sequences—specifically MK977832.1 for mutation sites 982, 989, 1007, 1011, and 1016 of the *Ae*. *aegypti vgsc* gene, NC_035109 for mutation site 410 of the *Ae*. *aegypti vgsc* gene, and KX371865.1 for mutation sites 1520, 1532, and 1534 of the *Ae*. *albopictus vgsc* gene using Geneious Prime 2024.0.5 (Dotmatics, Boston, MA, USA).

### 2.5. Synergist Bioassays for Malathion Metabolic Resistance Evaluation

Bioassays involving synergists were conducted using the CDC bottle bioassay method [42] (Figure S4) to evaluate metabolic resistance mechanisms to malathion in adult *Ae*. *albopictus* mosquitoes [43]. Approximately 125 adult female *Ae*. *albopictus* mosquitoes (F2) aged 3–5 days and unfed (no blood meal) were pre-exposed to three synergists: piperonyl butoxide (PBO, 400 μg/bottle) (PESTANAL^®^; Merck, Darmstadt, Germany), an inhibitor of cytochrome P450 monooxygenases; diethyl maleate (DEM, 80 µg/bottle) (Sigma-Aldrich, St. Louis, MO, USA), an inhibitor of glutathione S-transferases (GSTs); and S.S.S-tributylphosphotrithioate (DEF, 125 μg/bottle) (LGC Dr. Ehrenstorfer, Thermo Fisher Scientific, Augsburg, Germany), an inhibitor of esterase activity. Wheaton^®^ 250 mL bottles (Fisher Scientific) were coated with 1 mL of the CDC-recommended diagnostic concentration for each of these three synergists, while a control bottle was coated with 1 mL of acetone. Following a one hour pre-exposure to the synergist, the mosquitoes were immediately transferred to holding cages for a one-hour recovery period. At the end of this period, the mosquitoes were exposed to malathion at the CDC-recommended diagnostic dose (400 µg/bottle) for one hour. For each synergist condition, five bottles were prepared: four bottles each containing 25 mosquitoes (*n* = 100) intended for subsequent exposure to malathion, and one control bottle containing 25 mosquitoes exposed only to the synergist. Mortality was recorded every 5 min during the first 30 min of exposure, and then every 15 min until the end of the test. Mosquitoes were considered dead when they no longer responded to manual stimulation. After 30 min of exposure (the CDC diagnostic criterion), dead mosquitoes were classified as malathion-susceptible (MS). Mosquitoes still alive after one-hour of exposure in the bottle assay were classified as malathion-resistant (MR). Live and dead specimens were separated and placed individually in a 96-well plates, which were stored at −80 °C pending further analyses.

In parallel, a control test was conducted using the susceptible reference strain ATM-NJ95 (Figure S5). A total of 125 adult female *Ae*. *albopictus* mosquitoes (3–5 days old) that had not taken a blood meal were used and distributed among five Wheaton^®^ bottles (250 mL): four bottles (*n* = 100) were directly exposed to malathion at the CDC diagnostic dose (400 µg/bottle) for 1 h, without prior exposure to a synergist, while one control bottle was coated with acetone only (*n* = 25). Experimental conditions, mortality recording, and the classification of mosquitoes as susceptible or resistant to malathion were carried out in accordance with the CDC diagnostic criteria.

### 2.6. Statistical Analyses

Observed adult mosquito mortality following insecticide exposure was compared according to *kdr* genotype using Fisher’s exact test. Dose–response data for larvae were analyzed by probit regression to estimate lethal concentrations (LC_50_ and LC_95_) and resistance ratios (RR_50_) relative to the susceptible reference strain, in accordance with WHO guidelines. Hardy–Weinberg equilibrium was assessed for genotype frequencies. Mortality curves from bioassays involving synergists were generated using GraphPad Prism software v.10.5.0 (GraphPad, San Diego, CA, USA), and differences observed with or without pre-exposure to synergists were analyzed using Fisher’s exact test. The significance threshold was set at *p* < 0.05. All analyses were performed using R (version 4.4.0) and GraphPad Prism (version 10.5.0).

## 3. Results

### 3.1. Larval Susceptibility Assays

Dose–response curves were used to estimate the LC_50_ for *Ae*. *albopictus* larvae from Algiers exposed to temephos and *Bti* (Figure S6).

In parallel, the same experiments were conducted on larvae from the susceptible reference strain *Ae*. *albopictus* ATM-NJ95. For *Bti*, the LC_50_ was 30.3 µg/L and the LC_95_ was 77.4 µg/L for the Algiers population, compared to 52.3 µg/L and an LC_95_ of 117.1 µg/L for the reference strain, yielding a RR_50_ of 0.57 (Table 1). For temephos, the LC_50_ was 5.4 µg/L and the LC_95_ was 12 µg/L for the Algiers population compared to 3.2 µg/L and an LC_95_ of 8.2 µg/L for the susceptible strain ATM-NJ95, yielding to a RR_50_ of 1.35 (Table 1). According to the classification based on RR_50_ (RR_50_ < 5: susceptible; 5 ≤ RR_50_ ≤ 10: moderately resistant; RR_50_ > 10: resistant), these values indicate that the Algiers population remains fully susceptible to both larvicides, temephos and *Bti*.

### 3.2. Adult Susceptibility Assays

**Figure 2:**
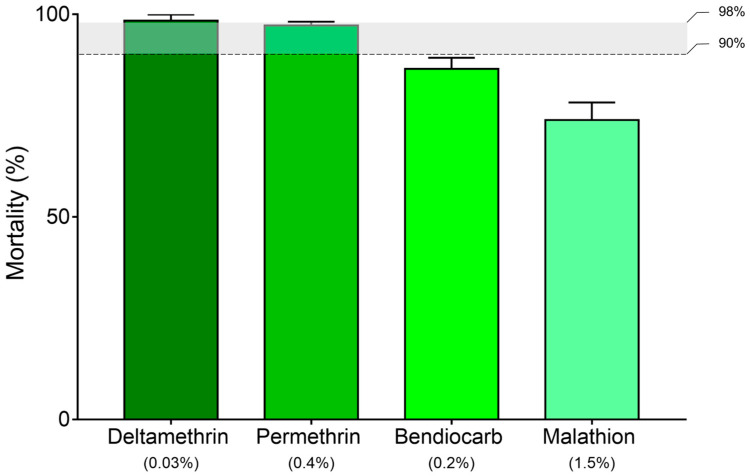
Mortality of *Aedes albopictus* in WHO tube bioassays after exposure to insecticides. The black hatched line represents the resistance threshold defined by the WHO (resistant if mortality ≤ 90%). The grey area between 90% and 98% of mortality indicates the suspected resistance zone. Above 98% mortality, the mosquito population is considered susceptible to the insecticide. The Error bars indicate standard deviation of the mean proportions.

Insecticide susceptibility tests were conducted exclusively on adult *Ae*. *albopictus* from the Algiers population. No mortality was observed after 24 h in the groups of mosquitoes exposed to control papers impregnated only with the corresponding solvent (i.e., silicone oil for pyrethroids and olive oil for organophosphates (malathion) and carbamates (bendiocarb)). However, variations in mortality rates were observed depending on the insecticides tested on *Ae*. *albopictus* mosquitoes from Algiers (Figure 2). For type II pyrethroids (i.e., 0.03% deltamethrin), the mortality rate for *Ae*. *albopictus* reached 98.4% (307/312), indicating full susceptibility to this compound, whereas for type I pyrethroids (i.e., 0.4% permethrin), a mortality rate of 97.2% (316/325) was recorded, suggesting possible resistance (i.e., suspected resistance). Regarding carbamates and organophosphates, mortality rates were 86.5% (275/318) for 0.2% bendiocarb and 73.7% (230/312) for 1.5% malathion, respectively, indicating resistance to both insecticides.

### 3.3. Characterization of Allele and Genotype Profiles of kdr Mutations Associated with Pyrethroid Resistance

All mosquitoes successfully genotyped exhibited a wild-type homozygous genotype (SS) for the *kdr* mutations V410L, L982W, S989P, A1007G, I1011V and T1520I (Table 2). In contrast, a low proportion of heterozygotes (SR) was detected for the three others three mutation sites, V1016G (5.9%), I1532T (7.4%), and F1534C/S (9.0%). No mosquitoes exhibited a resistant homozygous genotype (RR) at these loci. Allelic frequencies were 0.04 for the V1016G and I1532T mutations, and 0.05 for the F1534C/S mutation (Table 2).

None of the mutations studied showed a significant deviation from Hardy–Weinberg equilibrium, with all *p*-values exceeding 0.05. The distribution of phenotypes (live and dead individuals) according to multilocus *kdr* genotypes following exposure to deltamethrin and permethrin is illustrated in Figure S7 and Table S3.

For both insecticides, the wild-type multilocus genotype (VV/II/FF/) was the most frequent, accounting for 74.4% of individuals exposed to deltamethrin and 67.4% of those exposed to permethrin. Following exposure to deltamethrin, this genotype was predominantly associated with mortality, with only a small proportion of individuals surviving. A similar profile was observed following exposure to permethrin, although the number of survivors was slightly higher. Other multilocus genotypes were much less frequent (≤12.3%) and were primarily associated with mortality for both insecticides. Heterozygous genotypes at the V1016G, I1532T, and F1534C/S loci were also detected but showed no clear association with survival, as most individuals carrying these genotypes died after insecticide exposure. Combined analysis of the *kdr* loci I1532T and F1534C/S, performed at the individual level, revealed various genotypic profiles corresponding to wild-type homozygotes and simple heterozygotes. Notably, one mosquito carried both the F1534S and I1532T mutations, thereby constituting a double heterozygote (Table S4).

### 3.4. Impact of the Synergists DEF, DEM, and PBO on Malathion Resistance in Aedes albopictus from Algiers

To evaluate metabolic resistance to malathion, females from the Algiers population and the susceptible strain ATM-NJ95 were exposed to malathion in CDC bottles; mortality was recorded over time, as well as following pretreatment with the synergists DEF, DEM, or PBO.

**Figure 3:**
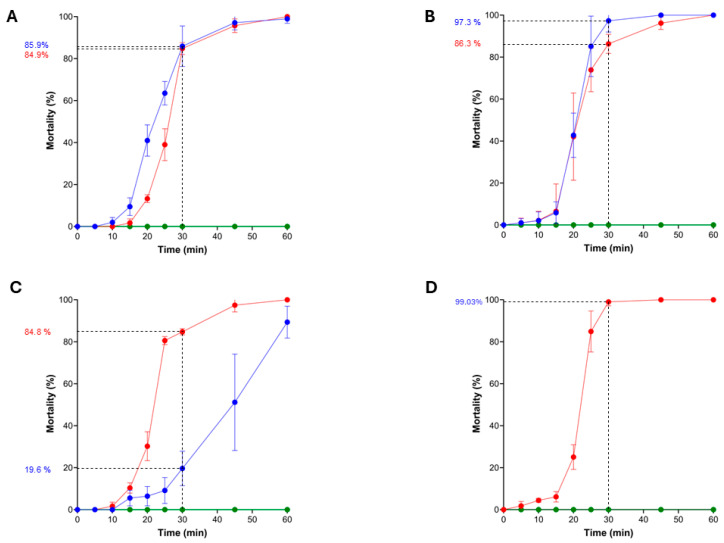
Mortality curves of *Aedes albopictus* without any exposure (control, green curves) or after pre-exposure to the synergists DEF, DEM, and PBO (blue curves) or without such (red curves), followed by 60 min of exposure to malathion in the CDC bottle bioassay: the dashed line indicates the mortality (%) at the diagnostic time to malathion in *Ae*. *albopictus* (30 min). (**A**) Pretreatment with DEM (diethyl maleate), (**B**) pretreatment with DEF (S.S.S-tributylphosphotrithioate), (**C**) pretreatment with PBO (piperonyl butoxide), and (**D**) susceptible reference strain ATM-NJ95.

In the presence of DEM (80 µg/mL), the mortality kinetics of the Algiers mosquitoes were similar to those observed with malathion alone. After 30 min of exposure, mortality rates were 84.9% with malathion alone and 85.9% following pretreatment with DEM and subsequent exposure to malathion, indicating that inhibition of the glutathione S-transferase (GST)-mediated detoxification pathway did not increase malathion toxicity (Figure 3A).

In mosquitoes of the Algiers strain pretreated with DEF (125 µg/mL), and subsequently exposed to malathion, an increase in mortality was observed, rising from 86.3% with malathion alone to 97.3% after 30 min of exposure (Figure 3B). The control group of Algiers mosquitoes, subjected to the same DEF pretreatment but exposed only to acetone, showed no mortality, thereby confirming the specific effect of malathion following DEF pretreatment. These results indicated that the esterase detoxification pathway is involved in malathion resistance within this population.

In contrast, pretreatment of mosquitoes with PBO (125 µg/mL) resulted in a marked decrease in mortality (19.6%) compared to mosquitoes exposed to malathion alone (84.8%) in the same experimental series (Figure 3C).

For the susceptible reference strain, *Ae*. *albopictus* ATM-NJ95, mortality was monitored following exposure in CDC bottles containing malathion at a concentration of 400 µg/mL. After 30 min, mortality reached 99.03% and after 35 min, all female were dead and unable to fly, consistent with the diagnostic times defined by the CDC protocol (Figure 3D). No mortality was observed in the unexposed controls. These results confirm the susceptibility of the reference strain and validate the experimental conditions used.

## 4. Discussion

The main findings of our study are as follows: (i) this is the first report on the phenotypic profile of baseline resistance in *Ae. albopictus* in Algiers, (ii) it is also the first molecular detection of *kdr* mutants known to be associated with resistance to pyrethroids, (iii) we have identified esterase-mediated resistance to malathion, and (iv) we have identified an unexpected antagonism between PBO and malathion. The results obtained in this study provide the first available data on the resistance of *Ae*. *albopictus* in Algiers, where the species has recently become established [7]. Larval bioassays revealed that the populations studied were susceptible to *Bti* and temephos, two biocides widely used in vector control programs. The resistance ratios (RR_50_) obtained for *Bti* and temephos, were below the threshold of 5, generally considered an indicator of susceptibility for the tested populations [40]. Becker et al. reported that in Germany, the exclusive and prolonged use of *Bti* for over 35 years did not lead to the emergence of resistance in *Ae*. *vexans*, despite large-scale applications [44]. Although mosquito larvae generally remain susceptible to *Bti*, some individuals can survive exposures that would otherwise be fatal. These larvae, exhibiting increased tolerance, can develop into adults whose biological characteristics—such as emergence rate, longevity, or fecundity—are altered by the residual effects of exposure during the larval stage [45].

In contrast, biological tests performed on adults revealed heterogeneous susceptibility profiles depending on the insecticide tested. The mosquitoes proved susceptible to deltamethrin, but probable resistance to permethrin (pyrethroids) as well as confirmed resistance to malathion (organophosphate) and bendiocarb (carbamate) were detected. The suspected resistance to permethrin, despite the persistence of susceptibility to deltamethrin, aligns with previous observations in *Aedes* and other mosquito vector, highlighting differences in susceptibility between type I and type II pyrethroids [46]. The differences in efficacy observed among insecticides can be attributed to several factors, notably variations in commercial formulations, differences in the metabolic pathways involved in molecules degradation, or the structural characteristics of the compounds—such as the α-cyano group present in type II pyrethroids, which modifies their interaction with voltage-gated sodium channels and, consequently, alters their toxic effect [47,48]. The observed resistance to malathion and bendiocarb fits into the broader context of multiresistance reported in *Aedes* populations from areas where different classes of insecticides are used simultaneously [27]. In Algiers, our results confirm the existence of a complex insecticide resistance profile and highlight the importance of regular, systematic monitoring to adapt vector control strategies accordingly.

Suspected permethrin resistance was further investigated by analyzing mutations in the *kdr* gene, which is known to be involved in pyrethroid resistance in mosquitoes. Our results revealed the presence of heterozygous mutant alleles (I1532T, F1534C, F1534S), as well as a double mutation at the F1534S and I1532T loci. This double-mutation genotype has previously been described as a resistance marker in several *Ae*. *albopictus* populations, primarily in China [49], and is considered a key element in the process of adaptation to pyrethroids. The presence of this mutation in Algiers populations suggests that its spread could result not only from local selection driven by insecticide pressure but also from the passive dispersal of mosquitoes via trade—a mechanism well documented in the global expansion of *Ae*. *albopictus* [50]. Continued use of permethrin in control interventions could exert strong selection pressure and accelerate the spread of these mutations, thereby increasing the risk of operational resistance becoming established and fixed in *Ae*. *albopictus* populations in Algiers.

The evaluation of metabolic mechanisms revealed the involvement of esterases in the observed resistance to malathion. A recent study [51] on malathion-resistant *Ae*. *albopictus* populations revealed, through a transcriptomic approach, strong induction of several detoxification-related genes, particularly those encoding esterases such as CCEae6a. These enzymes play a key role in the hydrolysis and neutralization of malathion, thereby reducing its insecticidal efficacy. The results showed that the expression of genes encoding esterases was significantly higher in resistant strains than in susceptible strains, confirming the major role of these enzymes in metabolic resistance mechanisms [51].

Other malathion synergists were evaluated to gain an overview of the metabolic mechanisms associated with resistance in *Ae*. *albopictus*. DEM (diethyl maleate), a glutathione S-transferases (GSTs) inhibitor, showed highlighted the limited role of GSTs in the observed resistance. In contrast, combining PBO (piperonyl butoxide)—a cytochrome P450 monooxygenase inhibitor—with malathion revealed an unexpected antagonism. The negative effect of PBO could be explained by metabolic interference or by the inhibition of malathion bioactivation pathways, as its insecticidal efficacy depends on its conversion into malaoxon [52,53,54]. Malaoxon, an oxidized and biologically active metabolite of malathion, can be metabolized by carboxylesterases. These enzymes play a key role in detoxification mechanisms by catalyzing the hydrolysis of malaoxon into more polar compounds, specifically mono- and dicarboxylic derivatives. These degradation products lack insecticidal activity, which reduces the efficacy of malathion. Consequently, increased carboxylesterase activity can contribute to resistance by reducing the amount of active compound capable of reaching its target [52,55]. A study conducted in Jamaica reported mortality rates ranging from 84% to 95% after 30 min of exposure to malathion, with 100% mortality reached after 45 min [56]. The authors suggest that this delay could be due to less efficient bioactivation of malathion into malaoxon, or possibly to slower cuticular penetration depending on the populations, although these mechanisms have not been directly demonstrated [56]. The results obtained did not allow us to determine whether cytochrome P450 enzymes were involved in malathion metabolism in this population. This assessment could be carried out through biochemical measurements of cytochrome P450s in resistant and susceptible *Ae*. *albopictus* individuals [57].

Although resistance to bendiocarb was observed in this study, tests with a synergist following the CDC protocol were not conducted for this insecticide. Indeed, the lack of standardized diagnostic doses and exposure times for bendiocarb limits the implementation and interpretation of such tests. Consequently, complementary approaches, such as biochemical or molecular analyses, would be required to better characterize the underlying resistance mechanisms [58].

In *Ae*. *albopictus*, resistance to bendiocarb appears to result from the involvement of multiple mechanisms, with a major contribution from detoxification processes [59]. Several studies have highlighted the role of cytochrome P450 enzymes, notably AalbCYP6Z8, which is capable of metabolizing carbamates such as bendiocarb [60]. Although carbamates are not commonly used in local mosquito control programs, resistance to bendiocarb can emerge through cross-resistance mechanisms. Recent studies have shown that the overexpression of carboxylesterases involved in organophosphates resistance can also confer resistance to carbamates—including bendiocarb in *Ae. aegypti*, *Anopheles gambiae s.l.* and *Ae. albopictus* [61]. Consequently, the resistance observed in the *Ae. albopictus* population from Algiers could result from selection pressure exerted by organophosphates such as malathion rather than from direct exposure to carbamates.

Furthermore, the potential role of cuticular resistance—linked to thickening or changes in cuticular composition [30] was not evaluated in this study. In *Ae*. *albopictus*, reduced insecticide susceptibility resulting from the overexpression of genes encoding cuticular proteins (leading to cuticle thickening) has been frequently reported [62,63]. Similarly, the role of the cytochrome P450 pathway was not directly examined. Additional analyses combining biochemical assays and microscopy techniques would make it possible to characterize these mechanisms and better understand the respective contributions of the cuticle and enzymes to organophosphate resistance.

The main limitations of the present study were as follows: (i) the observed antagonistic effect of piperonylbutoxide is consistent with the inhibition of malathion bioactivation pathways; however, further biochemical studies are required to confirm this mechanism; (ii) pooling *Ae. albopictus* specimens from the three different collection sites prior to bioassay precludes the assessment of spatial variability in resistance profiles and risk masking significant local differences in susceptibility. To truly determine whether our results are consistent across the sampled populations, a population genetic analysis will be necessary to verify whether or not *Ae. albopictus* populations in Algiers are structured.

This study provides an initial overview of insecticide resistance in *Ae. albopictus* in Algeria and serves as a baseline for monitoring changes in the species’ susceptibility over time. In the absence of existing data, these results will serve as a point of comparison for future surveillance activities and help document the evolution of resistance as *Ae. albopictus* continues to spread across Algeria [64]. From an operational standpoint, they also provide useful information to guide national vector control strategies. The first step should be to implement regular public awareness campaigns and educational initiatives aimed at the general public in order to limit the formation of *Ae. albopictus* breeding sites near residential areas. The second step should involve mechanically destroying breeding sites whenever possible, or using larvicides, as the *Ae. albopictus* populations in Algiers are susceptible to *Bti* and temephos. *Bti* is a particularly attractive option because of its high specificity toward mosquito larvae, its lack of toxicity to humans and other vertebrates, and its limited impact on non-target organisms when used in accordance with recommendations [65,66]. The final step is to use insecticides designed to target adults. In Algiers, resistance to malathion and bendiocarb, as well as suspected resistance to permethrin, necessitates a more judicious use of insecticides, particularly through the rotation of insecticides belonging to different chemical classes [40]. In this context, the repeated use of the same class of chemicals—particularly pyrethroids—should be limited in order to reduce the selection pressure that promotes the emergence and spread of resistance mechanisms [67]. The implementation of integrated resistance management strategies now appears essential to preserve the effectiveness of available insecticides and ensure the long-term sustainability of *Ae. albopictus* control programs in Algeria.

## 5. Conclusions

This study provides the first baseline profile of insecticide resistance in *Ae*. *albopictus* populations from Algiers. While larvicides remain effective, resistance to several adulticides—particularly organophosphates and carbamates—has already been observed, as well as the first signs of resistance to type I pyrethroids. The findings indicate that this resistance is driven by both metabolic mechanisms esterase activity and the early presence of *kdr* mutations. It also provides evidence-based data to support resistance surveillance, guide insecticide selection, and inform strategies for managing insecticide resistance within national vector control programs. Continuous resistance surveillance, combined with integrated vector management approaches, will be essential to preserve the effectiveness of available control tools and mitigate the public health risks associated with arbovirus outbreaks linked to the spread of this invasive vector.

## Figures and Tables

**Table 1:** Susceptibility profiles of larvae from *Aedes albopictus* colonies to *Bti* and temephos.

	*Bti*				Temephos			
*Ae. albopictus* Colony	LC_50_ (95% CI)	RR_50_ (95% CI)	LC_95_ (95% CI)	RR_95_ (95% CI)	LC_50_ (95% CI)	RR_50_ (95% CI)	LC_95_ (95% CI)	RR_95_ (95% CI)
ATM-NJ95	52.3 (11.5–161.5)	1	117.1 (30.2–290.4)	1	3.2 (0.2–63.6)	1	8.2 (0.04–1845.0)	1
Algiers	30.3 (6.6–139.2)	0.57 (0.55–0.60)	77.4 (5.0–1194.1)	0.71 (0.65–0.78)	5.4 (0.8–37.4)	1.35 (1.14–1.59)	12 (0.4–385.4)	1.28 (1.27–1.30)

LC_50_: Lethal concentration required to kill 50% of the specimens tested. LC_95_: Lethal concentration required to kill 95% of the specimens tested. RR: Resistance Ratio; CI: Confidence Interval. LC_50_ and LC_95_ are expressed in µg/L.

**Table 2:** Allelic and genotypic frequencies of *kdr* mutations in *Aedes albopictus* mosquitoes from Algiers, Algeria.

*kdr* Alleles	*n* (%)				Frequency ^1^	χ^2^ HWE	*p*-Value
	SS	SR	RR	UI			
V410L	186 (98.9)	0 (0)	0 (0)	2 (1.1)	0	NA	NA
L982W	88 (46.8)	0 (0)	0 (0)	100 (53.2)	0	NA	NA
S989P	137 (72.9)	0 (0)	0 (0)	51 (27.1)	0	NA	NA
A1007G	136 (72.3)	0 (0)	0 (0)	52 (27.7)	0	NA	NA
I1011V	137 (72.9)	0 (0)	0 (0)	51 (27.1)	0	NA	NA
V1016G	116 (61.7)	11 (5.9)	0 (0)	61 (32.4)	0.04	0.26	0.61
T1520I	169 (89.9)	0 (0)	0 (0)	19 (10.1)	0	NA	NA
I1532T	155 (82.5)	14 (7.4)	0 (0)	19 (10.1)	0.04	0.32	0.57
F1534C/S	153 (81.4)	17 (9.0) *	0 (0)	18 (9.6)	0.05	0.47	0.63

^1^ Frequency of mutant allele; S, susceptible; R, resistant; UI, uninterpretable; *kdr*, knockdown resistance; HWE, Hardy–Weinberg equilibrium at 5% significance level; NA, not applicable. * (For F1534C/S, the total mutant allele count (17) includes F1534C (*n* = 5) and F1534S (*n* = 12)).

## Data Availability

All data sets are included in this published article and its Supplementary Information files.

## Supplementary Materials

The following supporting information can be downloaded at: https://www.mdpi.com/article/10.3390/insects17070696/s1, Figure S1. Photography of the environment of the collection site I in El-Harrach district. Figure S2. Schematic representation of the experimental protocol used to determine the susceptibility of *Aedes albopictus* larvae to temephos and *Bacillus thuringiensis israelensis* (Bti). Figure S3. Schematic representation of the WHO tube bioassay, including the test components and the main steps of the experimental procedure. Figure S4. Diagram illustrating the protocol for conducting the CDC’s bottle bioassay with synergists agents to evaluate insecticide resistance in *Aedes albopictus*. Figure S5. Diagram illustrating the CDC’s bottle bioassay protocol for evaluating resistance to malathion in the susceptible reference strain of *Aedes albopictus* (ATM-NJ95). Figure S6. Dose–response curves of *Aedes albopictus* larvae exposed to temephos and *Bacillus thuringiensis israelensis* (*Bti*): the responses of *Ae*. *albopictus* larvae from the Algiers population (A, C) and the susceptible ATM-NJ95 reference strain (B, D) exposed to *Bti* (A, B) and temephos (C, D) are shown. The curves represent larval mortality as a function of increasing concentrations of each larvicide after one hour exposure, based on WHO insecticide susceptibility assays. The dotted lines indicate the estimated lethal doses (LD_25_, LD_50_, LD_95_). Figure S7. Distribution of multilocus haplotypes (*kdr* mutations V1016G, I1532T and F1534C/S) in dead and surviving *Aedes albopictus* mosquitoes exposed to pyrethroids in Algiers, Algeria: stacked bar plots represent the proportion of alive (green) and dead (red) mosquitoes for each multilocus genotype group after exposure to Deltamethrin (A) or Permethrin (B). Genotypes correspond to the different combinations of the V1016G, I1532T and F1534C/S mutations. Table S1: Sampling sites and GPS coordinates for the collection of *Aedes albopictus* eggs; Table S2: Primer sequences and concentrations used for amplification of *vgsc* gene fragments targeting specific mutations; Table S3: Genotypes and their association with pyrethroid resistance in *Aedes albopictus* from Algiers, Algeria. Table S4: Combined genotypic distribution at the *kdr* loci I1532T and F1534C/S in the studied mosquito population.

## Abbreviations

The following abbreviations are used in this manuscript:

*Bti*	*Bacillus thuringiensis israelensis*
CDC	Centres for Disease Control and Prevention
CI	Confidence interval
DEF	S,S,S-tributyl phosphorotrithioate (esterase inhibitor)
DEM	Diethyl maleate (glutathione S-transferase inhibitor)
DNA	Deoxyribonucleic acid
EFS	French Blood Establishment (Établissement Français du Sang)
F0, F1, F2	Filial generations
GST	Glutathione S-transferase
*kdr*	Knockdown resistance
LC_50_	Lethal concentration killing 50% of exposed individuals
LC_95_	Lethal concentration killing 95% of exposed individuals
MS	Malathion-susceptible
MR	Malathion-resistant
PCR	Polymerase chain reaction
PBO	Piperonyl butoxide (cytochrome P450 inhibitor)
RR_50_	Resistance ratio at LC_50_
RR_95_	Resistance ratio at LC_95_
*vgsc*	Voltage-gated sodium channel
WHO	World Health Organization
