# Supplementary material for: Evaluation of Insecticide Resistance in Aedes albopictus Population from Algiers, Algeria
Source: Insects. 2026 Jul 4;17(7):696. doi: 10.3390/insects17070696 (PMC13411700; doi:10.3390/insects17070696)
Supplement: Supplementary file 1 [file insects-17-00696-s001.zip › insects-4370779-supplementary/Figure S2.pdf]

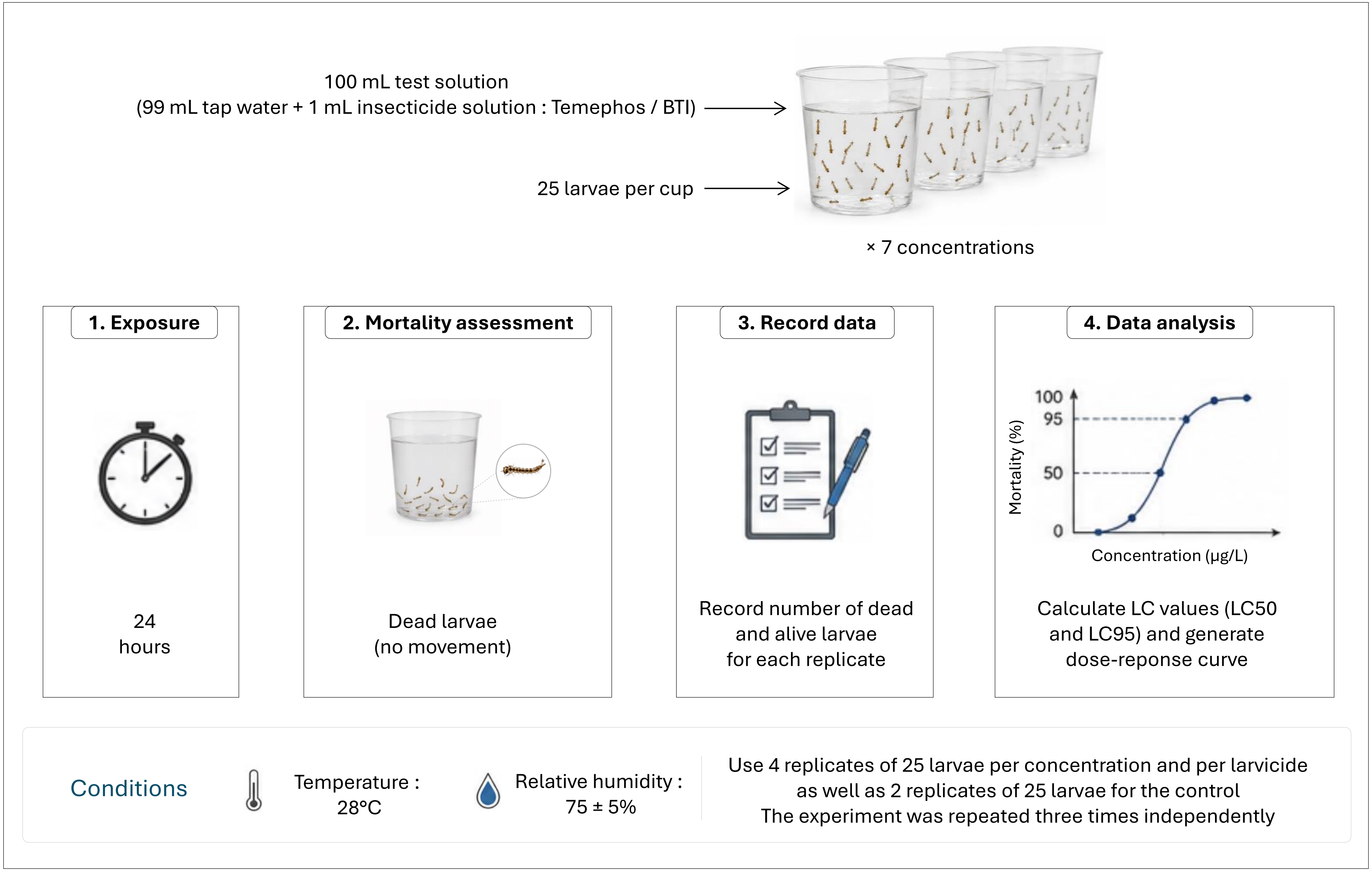
100 mL test solution
(99 mL tap water + 1 mL insecticide solution : Temephos / BTI)
25 larvae per cup
× 7 concentrations
1. Exposure
24
hours
2. Mortality assessment
Dead larvae
(no movement)
3. Record data
Record number of dead
and alive larvae
for each replicate
4. Data analysis
100
95
50
0
Mortality (%)
Concentration (µg/L)
Calculate LC values (LC50
and LC95) and generate
dose-reponse curve
Conditions
Temperature :
28°C
Relative humidity :
75 ± 5%
Use 4 replicates of 25 larvae per concentration and per larvicide
as well as 2 replicates of 25 larvae for the control
The experiment was repeated three times independently
