# Supplementary material for: Evaluation of Insecticide Resistance in Aedes albopictus Population from Algiers, Algeria
Source: Insects. 2026 Jul 4;17(7):696. doi: 10.3390/insects17070696 (PMC13411700; doi:10.3390/insects17070696)
Supplement: Supplementary file 1 [file insects-17-00696-s001.zip › insects-4370779-supplementary/Figure S3.pdf]

1. Individual components

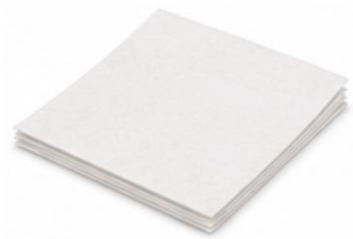

Insecticide-impregnated paper
lined inside the exposure tube

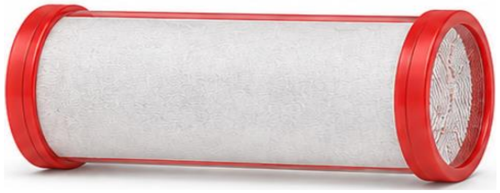

Exposure tube inner wall lined with
Insecticide-impregnated paper

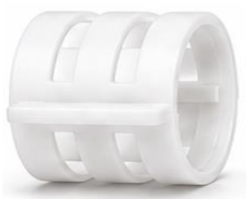

Connector clip
Joins the two tubes

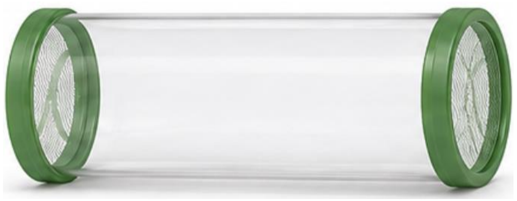

Holding tube
Holds mosquitoes after exposure

2. Bioassay procedure (WHO standard)

1. Exposure

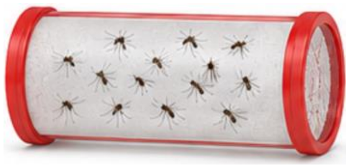

Introduce 25 mosquitoes into the exposure tube **for 1 hour**

2. Transfer

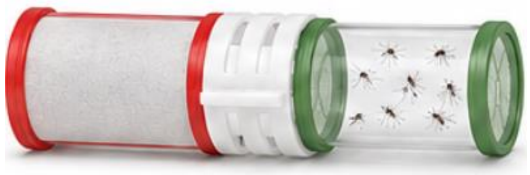

After 1 hour, transfer mosquitoes into the holding tube through the connector

3. Holding

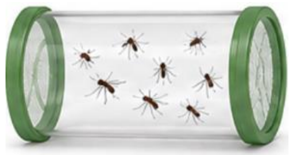

Hold mosquitoes under standard conditions **for 24 hours**

4. Mortality Assessment

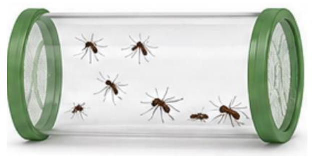

Record the number of dead and alive mosquitoes **(mortality)**

Conditions	Temperature : 28°C	Relative humidity : 75 ± 5%	Use 4 replicates of at least 25 mosquitoes per insecticide and 2 replicates of at least 25 mosquitoes for control
