# Supplementary material for: Evaluation of Insecticide Resistance in Aedes albopictus Population from Algiers, Algeria
Source: Insects. 2026 Jul 4;17(7):696. doi: 10.3390/insects17070696 (PMC13411700; doi:10.3390/insects17070696)
Supplement: Supplementary file 1 [file insects-17-00696-s001.zip › insects-4370779-supplementary/Figure S4.pdf]

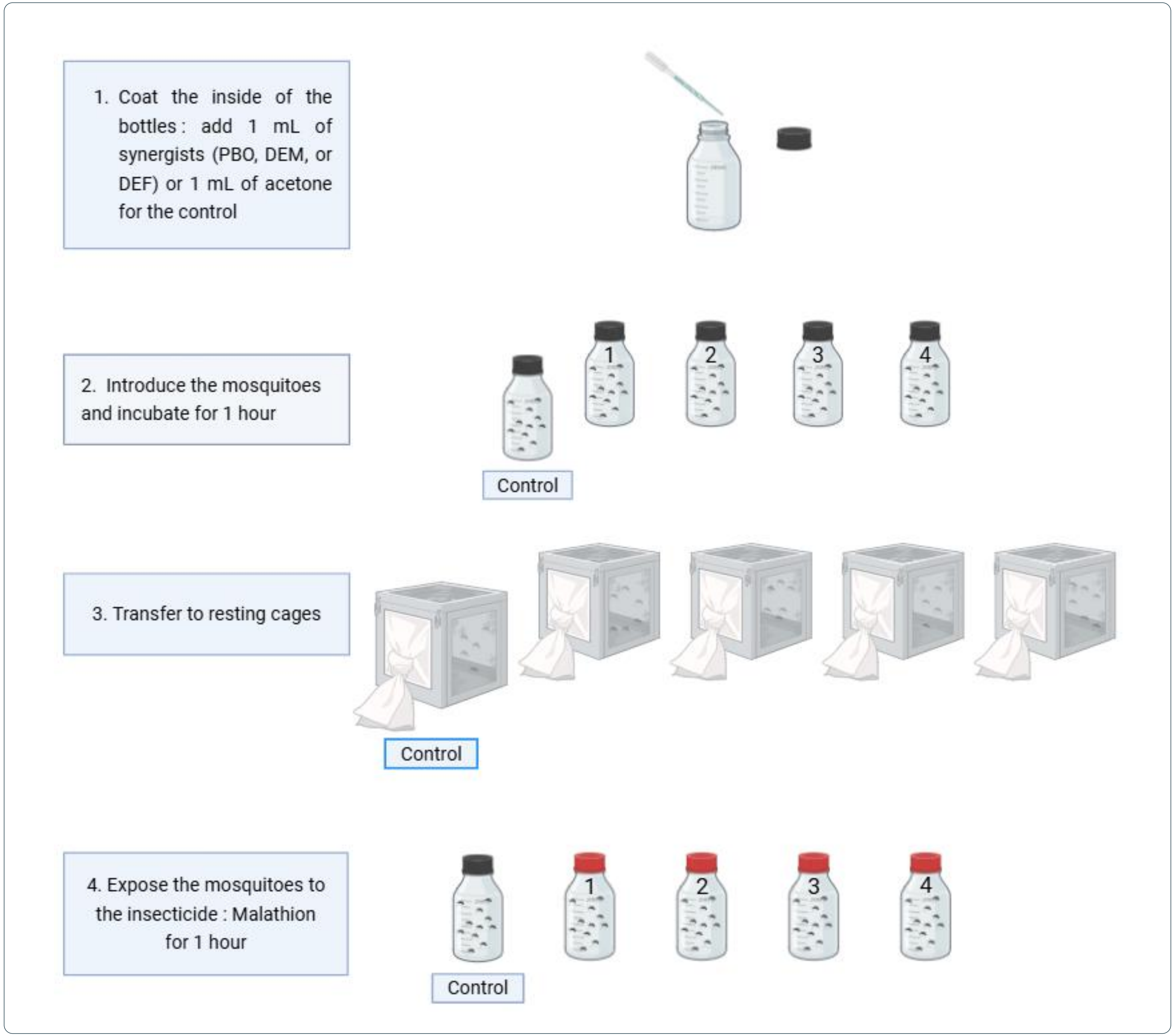
1. Coat the inside of the bottles: add 1 mL of synergists (PBO, DEM, or DEF) or 1 mL of acetone for the control
2. Introduce the mosquitoes and incubate for 1 hour
Control
1
2
3
4
3. Transfer to resting cages
Control
4. Expose the mosquitoes to the insecticide : Malathion for 1 hour
Control
1
2
3
4
