# Supplementary material for: Evaluation of Insecticide Resistance in Aedes albopictus Population from Algiers, Algeria
Source: Insects. 2026 Jul 4;17(7):696. doi: 10.3390/insects17070696 (PMC13411700; doi:10.3390/insects17070696)
Supplement: Supplementary file 1 [file insects-17-00696-s001.zip › insects-4370779-supplementary/Table S1.pdf]

Table S1. Sampling sites and GPS coordinates for the collection of *Aedes albopictus* eggs.

Site N°	Sites name	Wilaya	District	Type of habitat	Collection period	GPS coordinates
**1**	S	Algiers	Bir Mourad Rais	Urban	September–October 2024	36°44'05.0"N 3°03'27.4"E
**2**	H	Algiers	Hussein-Dey	Urban	September–October 2024	36°43'58.9"N 3°06'45.8"E
**3**	I	Algiers	El-Harrach	Urban	September–October 2024	36°43'15.3"N 3°08'57.0"E

Three urban sites in Algiers, Algeria, were selected for ovitrap-based egg collection. The table lists site number, name, administrative location, habitat type, period of collection and GPS coordinates, providing the geographical context for the field-collected mosquito populations used in this study.
