# Supplementary material for: Evaluation of Insecticide Resistance in Aedes albopictus Population from Algiers, Algeria
Source: Insects. 2026 Jul 4;17(7):696. doi: 10.3390/insects17070696 (PMC13411700; doi:10.3390/insects17070696)
Supplement: Supplementary file 1 [file insects-17-00696-s001.zip › insects-4370779-supplementary/Table S2.pdf]

Table S2. Primer sequences and concentrations used for amplification of *vgsc* gene fragments targeting specific mutations.

Codon Mutation[#]	5′→3′ Sequences	PCR product (bp)	PCR conditions*	Final concentration (μM)
V410L	F_ATTATCCCCACTCTCCCCCT<br>R_TTGCACACATACACACACGG	162	95/62/72	0.625
L982W, S989P, A1007G, I1011V, V1016G	F_CTGCCACGGTGGAACTTCA<br>R_TTGTTCGTTTCGTTGTCGGC	478	95/59/72	0.25
T1520I, I1532T, F1534C/S	F_GTGGGAAAGCAGCCGATTC<br>R_CCTAGGCCGTGGAATAGCTT	258	95/59/72	0.375

[#]When multiple mutations are listed for the same primer pair, they correspond to substitutions located within the same amplified *vgsc* fragment. F1534C/S indicates the detection of alternative substitutions at the same codon with the same primer pair. *PCR conditions are expressed as denaturation / annealing / extension temperatures (°C). bp, base pairs; F, forward; R, reverse; *vgsc*, voltage-gated sodium channel.
