# Supplementary material for: Evaluation of Insecticide Resistance in Aedes albopictus Population from Algiers, Algeria
Source: Insects. 2026 Jul 4;17(7):696. doi: 10.3390/insects17070696 (PMC13411700; doi:10.3390/insects17070696)
Supplement: Supplementary file 1 [file insects-17-00696-s001.zip › insects-4370779-supplementary/Table S3.pdf]

Table S3. Genotypes and their association with pyrethroid resistance in *Aedes albopictus* from Algiers, Algeria.

Insecticides	Genotypes	Number	Frequency (%)	Phenotypes		*p*-value[1]
				Dead	Alive	
Deltamethrin	VV /II / FF	46	74.4	43	3	Reference
	VG / II / FF	2	3.3	1	1	0.16
	VV / IT / FF	5	8.2	5	0	>0.99
	VV / II / FC	4	4.9	4	0	>0.99
	VV / II / FS	4	6.6	4	0	>0.99
Permethrin	VV /II / FF	33	67.4	28	5	Reference
	VG / II / FF	6	12.2	4	2	0.29
	VV / IT / FF	5	10.2	5	0	>0.99
	VV / II / FC	1	2.0	0	1	0.18
	VV / II / FS	4	8.2	4	0	>0.99

[1]Fisher exact test was used to assess the association between genotypes and phenotypes (dead vs alive), using the wild-type genotype (VV / II / FF) as the reference group. No significant association was detected between the tested genotypes and survival after exposure to deltamethrin or permethrin ($p > 0.05$).
