# Supplementary material for: Evaluation of Insecticide Resistance in Aedes albopictus Population from Algiers, Algeria
Source: Insects. 2026 Jul 4;17(7):696. doi: 10.3390/insects17070696 (PMC13411700; doi:10.3390/insects17070696)
Supplement: Supplementary file 1 [file insects-17-00696-s001.zip › insects-4370779-supplementary/Table S4.pdf]

Table S4. Combined genotypic distribution at the *kdr* loci I1532T and F1534C/S in the studied mosquito population.

		I1532T	
		SS	SR
F1534C/S	SS	139	13
	SR	16	1

SS, homozygous susceptible; SR, heterozygous; *kdr*, knockdown resistance. Each cell shows the number of individuals carrying the corresponding combination of genotypes at the two loci. For F1534C/S, the SR category includes individuals carrying either F1534C or F1534S mutations.
