# Supplementary figures and images for: Evaluation of Insecticide Resistance in Aedes albopictus Population from Algiers, Algeria
Source: Insects. 2026 Jul 4;17(7):696. doi: 10.3390/insects17070696 (PMC13411700; doi:10.3390/insects17070696)

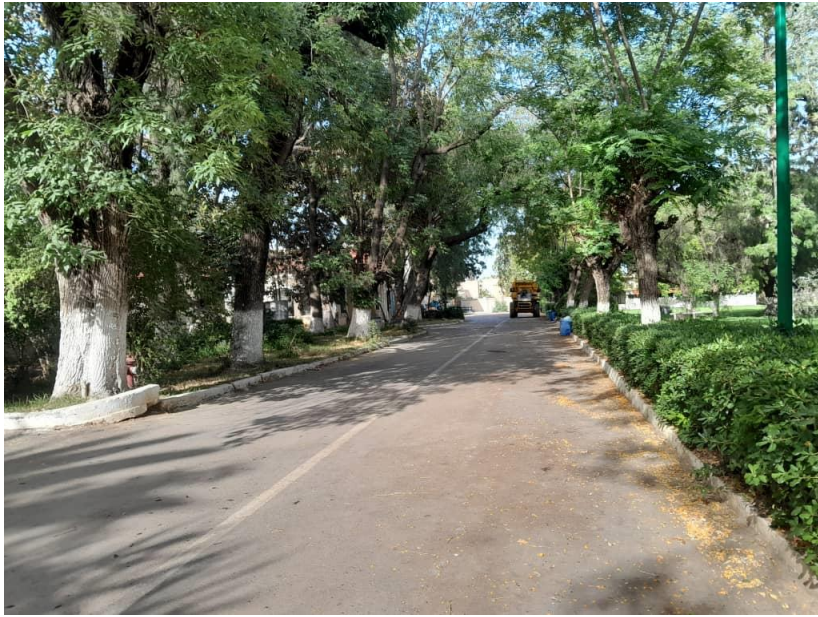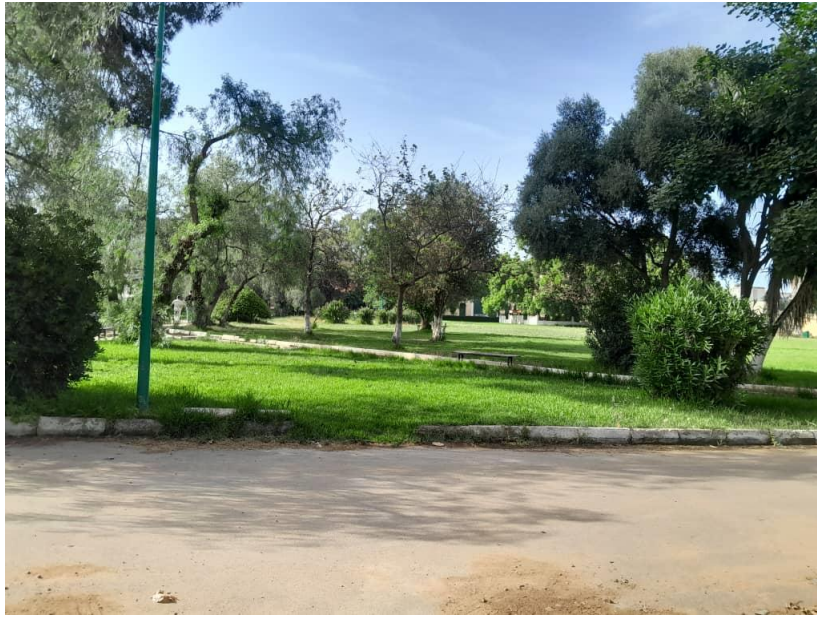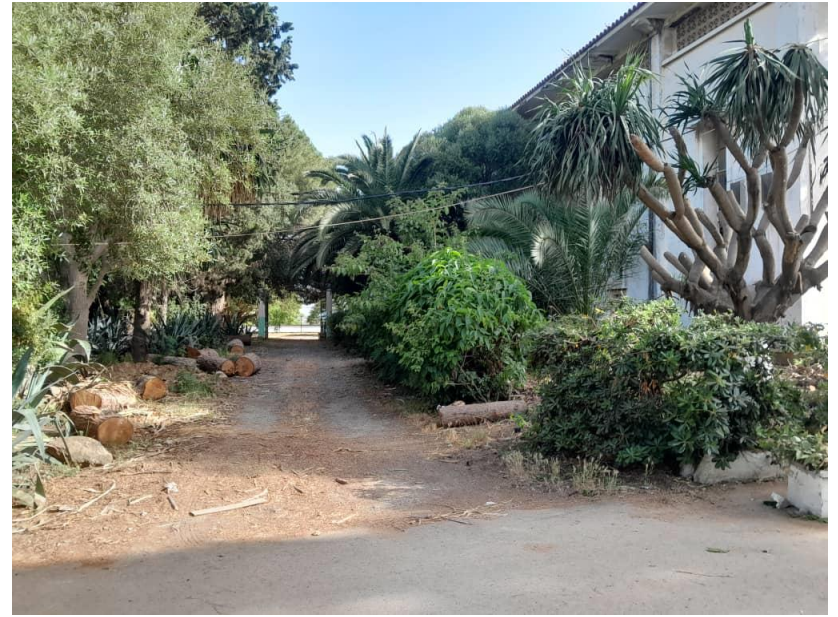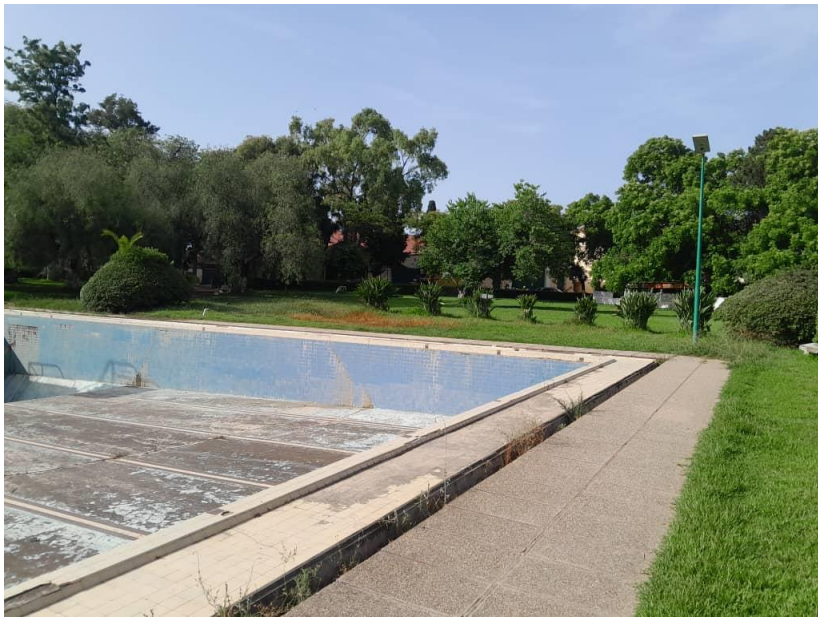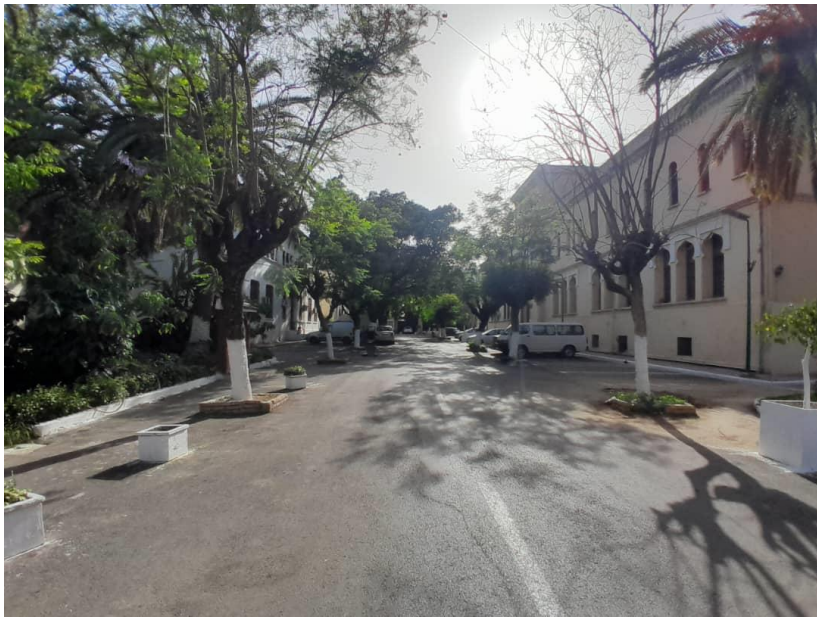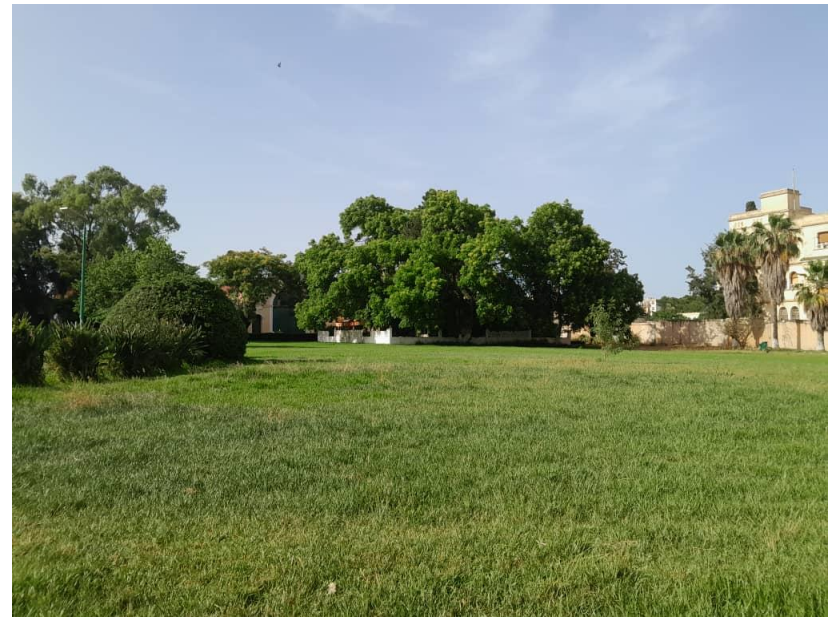

Supplement: Supplementary file 1 [file insects-17-00696-s001.zip › insects-4370779-supplementary/Figure S1.pdf]

A

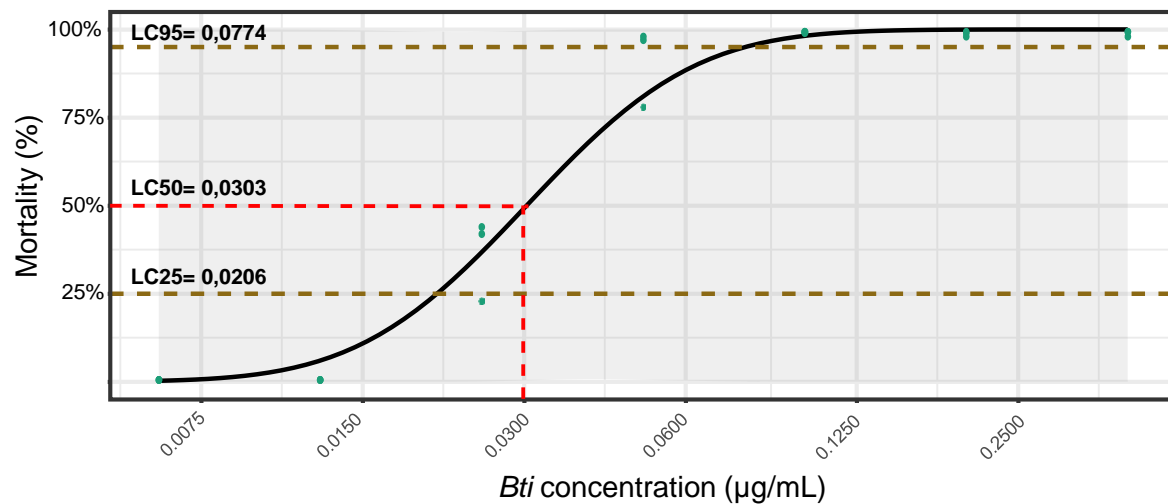

B

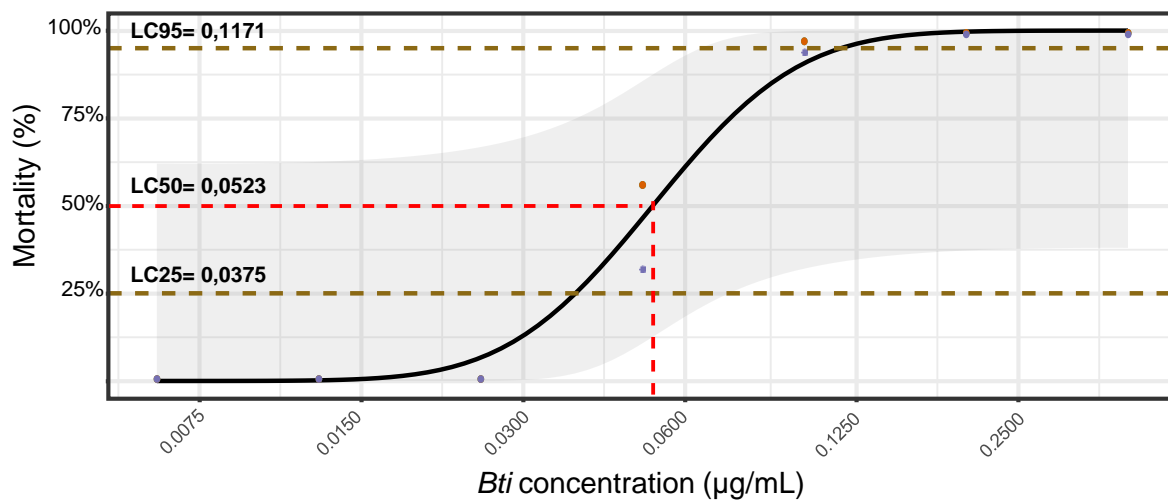

C

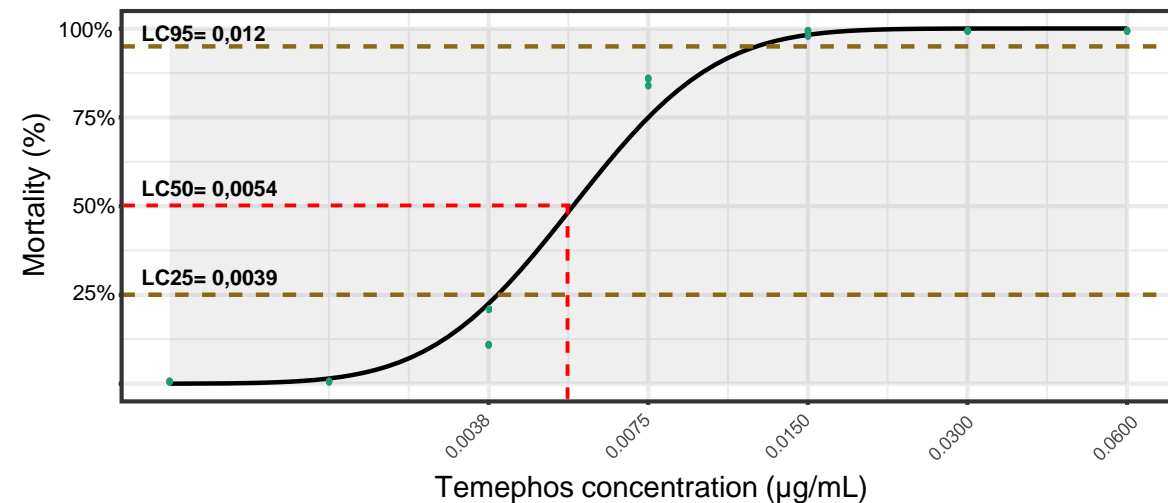

D

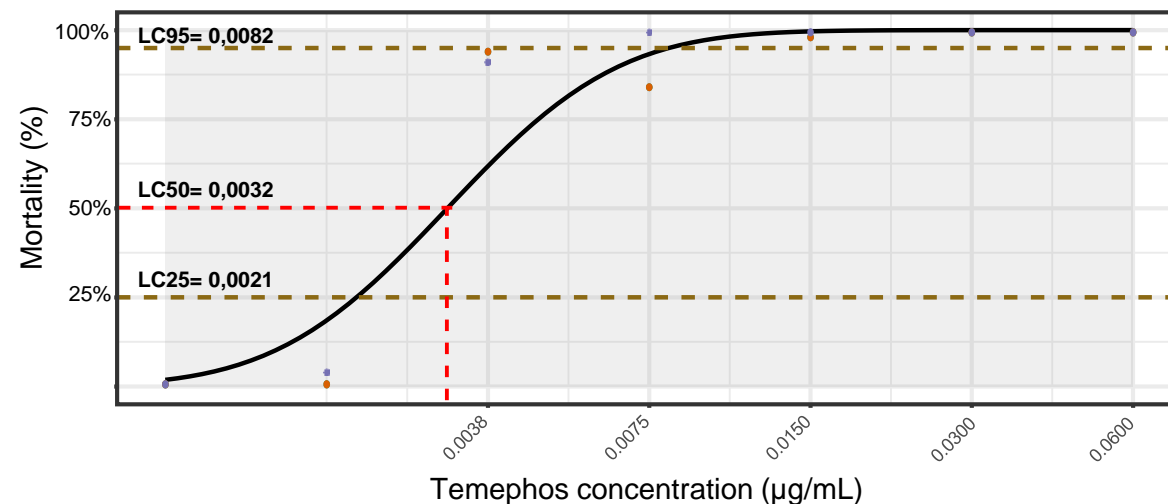

Supplement: Supplementary file 1 [file insects-17-00696-s001.zip › insects-4370779-supplementary/Figure S6.pdf]

A

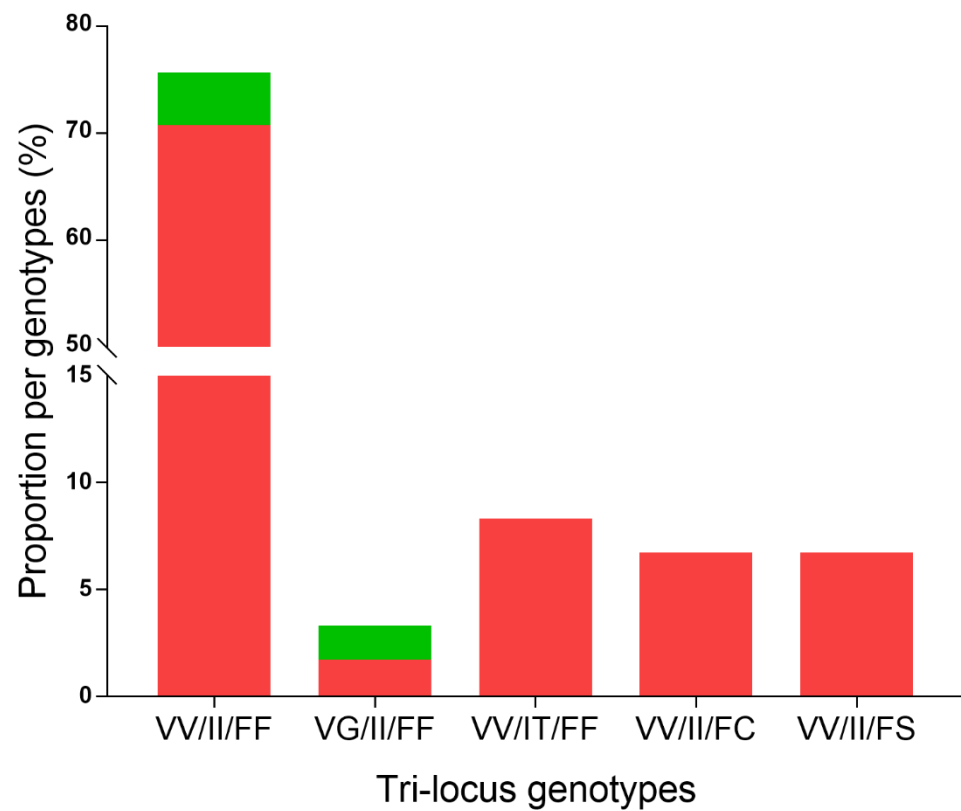

B

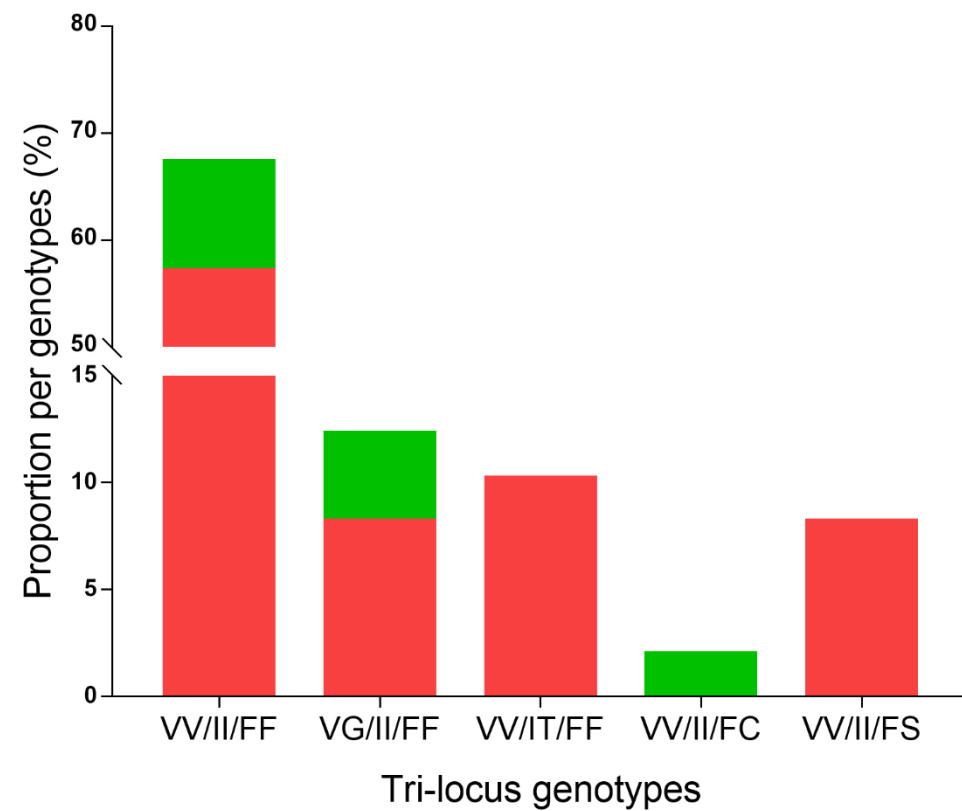

Supplement: Supplementary file 1 [file insects-17-00696-s001.zip › insects-4370779-supplementary/Figure S7.pdf]
